# Editorial: Regulation of permeability of tight junctions

**DOI:** 10.3389/fcell.2026.1816392

**Published:** 2026-03-05

**Authors:** Claudia Tanja Mierke

**Affiliations:** Faculty of Physics and Earth System Science, Peter Debye Institute of Soft Matter Physics, Biological Physics Division, Leipzig University, Leipzig, Germany

**Keywords:** dietary food components, gastrointestinal barrier, *Helicobacter pylori*, mast cells, occludin, probiotics, tight junctions (TJs), ZO-1

In the field of cell and developmental biology, the Research Topic “Regulation of tight junction (TJs) permeability” is an emerging area of research. Special emphasis is placed on the selective barrier function of epithelial cell layers of the intestinal tract or pulmonary system. Although research activities in this specialized field have increased significantly, there are still serious gaps in research that need to be filled immediately. Key gaps in research on the control of TJ permeability comprise the limited understanding of the effects of dietary nutrients, failure to validate *in vitro* findings *in vivo*, and the requirement for more specific, non-destructive treatment approaches to re-establish the barrier function. Even though inflammatory reactions have been seen, the specific impact of dietary substances like fatty acids, phytochemicals, and nutrients on modulating TJ permeability is still not totally clear, so a gap exists in the comprehension of how diet can impact barrier performance. It is also necessary to differentiate between and selectively target specific paracellular pathway types, such as the “pore route” for small ions and water versus the “leakage route” for larger molecules instead of measuring only the total, non-selective, injury-related fluid flow. The specific mechanisms through which adhesion junctions (AJs) and gap junctions (GJs) communicate with each other and control the integrity of the TJ barrier continue to be an intriguing but still relatively little-studied area.

Moreover, the barrier function of epithelial tissues plays a crucial role both from a physiological perspective and in disease patterns like gastrointestinal or airway disorders. Cell-cell adhesions such as TJs are essential for maintaining the equilibrium of absorption and secretion mechanisms inside the gastrointestinal tract, while simultaneously preventing the penetration of harmful luminal material. The barrier also conveys the immune system’s response to potential foreign invaders such as bacteria like *Helicobacter pylori* or viruses, antigens and damaging substances such as molecules from food, while allowing valuable nutrients to pass through or be transported into the bloodstream. Moreover, the protective effect of probiotics for the functionality of the TJ-facilitated barrier function seems to be critical. In addition, a dietary fatty acid overload can alter the mitochondria functions and subsequently the barrier function of the gastrointestinal tract. Specifically, TJs control the paracellular transportation of water, nutritional substances, and ions. Due to this specific regulation of the gastrointestinal barrier, TJs are an integral part of controlling epithelial polarity and paracellular permeability (e.g. porosity). These TJs are supported by a complex mesh of protein linkages made up of peripheral membrane adapter proteins like zonula occludens-1 (ZO-1), ZO-2, and ZO-3. They are multi-domain scaffolding proteins that localize to the cytoplasmic surface of the TJ and serve as the primary connector between transmembrane proteins such as occludin, claudins, junctional adhesion molecule (JAM), and tricellulin on the one hand and the actin cytoskeleton on the other. Occludin, a key regulatory molecule of the Tight Junction-associated MARVEL Protein (TAMP) family, controls the formation of junctions and signal transduction. Phosphorylation of occludin is the primary regulatory mechanism. Enhanced phosphorylation of specific serine/threonine residues, like Ser-507 and Thr-382, typically reinforces the strength of the barrier, whereas dephosphorylation or tyrosine phosphorylation, which is frequently carried out by Src kinases, leads to the breakdown of TJs and evelavted permeability. The OCEL (C-terminal domain) is important for connecting with ZO-1 and reacting to cytokines like TNF-*α* that change the function of the barrier (Yuan et al., 2020). Claudins are regarded as the most important, characteristic structural framework of TJ networks. Junctional adhesion molecules (JAMs) are Ig-like proteins like JAM-A that contribute to the development of the junction complex and, together with claudins, control the “leakage route”. Tricellulin accumulates in specific areas where 3 cells intersect (tri-cell junctions) and effectively acts as a sealant at these specific intersection points.

This Research Topic aims to investigate the impact of dietary nutrients on the control of TJ permeability. Examining these effects should improve the understanding of how dietary consumption can affect the permeability of TJs to small polar compounds, such as phenolic antioxidants and low molecular weight compounds, and impact inflammatory processes and associated diseases. The ultimate goal is to gain new insights that may result in more effective treatment options for diseases linked to the malfunctioning of TJs.

The Research Topic comprises seven articles, two reviews and five original experimental articles. The first review article by Ferris et al. deals with the effect of probiotics on the intestinal TJ barrier function that are hypothesized to possess a therapeutic effect on a multiple gastrointestinal (GI) disorders, such as inflammatory bowel disease. The review article points out that certain strains such as *Lactobacillus*, Bifidobacterium, *Escherichia coli* Nissle 1917, *Bacillus subtilis*, and *Saccharomyces* *boulardii* can increase the strength of the TJ barrier in in vitro cell culture models, in *in vivo* animal models, and in some clinical situations. These probiotics can act through various mechanisms, among them the upregulation of TJ proteins like occludin, claudins, and ZO-1, downregulation of proinflammatory cytokines, suppression of NF-κB, myosin light chain kinase (MLCK), and mitogen-activated protein kinases (MAPK) signal transduction pathways, and stimulation of host pattern recognition receptors including toll-like-rezeptor 2 (TLR-2) and peroxisome poliferator-activated receptor gamma (PPARγ). These effects appear to be strain-specific, which leads to the consideration of identifying and characterizing single probiotic strains for use in therapy. Finally, the results discussed reinforce the potential of probiotics as complementary or prophylactic treatments targeting epithelial barrier dysfunction across a variety of GI disorders. Nevertheless, the findings can have the potential to be utilized as personalized treatments. This review sheds light on both the promising potential and the complex nature of probiotic-driven gut barrier control, offering valuable insights for future research.

The second review article by Ryan and Braydon provides an overview of the control of the intestinal permeability of epithelial TJs in the small intestine by molecules originating from food. As a result, nutrient absorption, osmotic balance in the lumen, and paracellular invasion of pathogens can be strengthened. Medium-chain fatty acids and derivatives can temporarily and reversibly open epithelial TJs to enable therapeutic peptides to be administered orally. Other active ingredients obtained from food, such as chitosan, improve mucosal adhesion and can also influence TJ permeability in the gut. Pelargonidin, an isolated polyphenol pigment from strawberries, has been found to be a prospective TJ opener that enhances oral insulin administration in rats. In contrast, short-chain fatty acid (SCFA), sodium butyrate, several essential/non-essential amino acids, fermented food, zinc and anthocyanins exert antioxidant effects and hence reinforce the integrity of TJs. Consequently, this offers potential therapeutic advantages for diseases associated with elevated intestinal permeability.

The first original research article by Romero-Fabela et al. explores the disruptive nature of *H. pylori* cytotoxin-associated gene A (CagA) on the integrity of the pancreatic epithelial barrier. The oncoprotein CagA plays the most important role in the development of stomach diseases such as pancreatic cancer caused by infection with *H. pylori* CagA+. Nevertheless, the exact mechanisms are uncertain. They infected BxPC-3 pancreatic epithelial cells with *H. pylori* bacterial strains that were either CagA+ or CagA− for the purpose of analyzing the precise mechanism. Infection with CagA+ strains caused elevated release of IL-8 and significant disturbance of the integrity of the epithelial barrier, as evidenced by transepithelial electrical resistance tests. In line with these findings, altered expression patterns were observed, such as increased concentrations of claudin-4 and occludin and decreased concentrations of E-cadherin and β-catenin, as well as cytoplasmic relocation of important proteins of the apical junction complex. In addition, there was a restructuring of the cytoskeleton, such as the absence of the apical actin ring and a displacement of cortactin and vinculin on the periphery. Both changes are accompanied by alterations in shape and elevated cell motility. Consequently, CagA appears to alter the performance of pancreatic epithelial cells, thereby acting as a risk factor for the pathogenesis of H. pylori-associated pancreatic diseases.

The second original research article by Hou et al. examines the restoration via electroacupuncture (EA) of the intestinal mucosal barrier in irritable bowel syndrome with diarrhea (IBS-D) rats. This alters the exosomal microRNAs (MC-EXO miRNAs) originating from mast cells, such as miR-149-5p, which is linked to cancer biology and intestinal barrier function, and miR-22-5p, which controls heart, lung, and liver function. Moreover, the intestinal barrier dysfunction in IBS-D is attributable to mast cells via tryptase release. EA suppresses mast cell activation, CRF and CRF-R1 expression and enhances TJ protein expression, resulting in decreased intestinal permeability and improved barrier restoration. These beneficial effects were noticeably reduced by corticotropin-releasing factor (CRF) or mast cell agonists, indicating that EA is caused by blocking CRF/CRF-R1 and activating mast cells. In the model group, the expression levels of miR-149-5p and miR-22-5p (enriched in TJ-related signaling pathways) are significantly higher than in the EA group. Transfection of Caco-2 cells with adeno-associated viruses carrying miR-149-5p or miR-22-5p analogues enhanced the permeability of the monolayer and decreased the levels of TJ proteins. In addition, miR-149-5p reversed the protective impact of EA in IBS-D rats, implying that miR-149-5p affects EA-mediated restoration of the gut barrier.

The third original research article by Guerbette et al. showed that saturated fatty acids have varying effects on mitochondrial function and the intestinal epithelial barrier in the *in vitro* model of IPEC-J2 enterocytes, according to their chain length. Intestinal barrier integrity is primarily dependent on the mitochondrial function of intestinal epithelial cells, which generate ATP via oxidative phosphorylation (OXPHOS). There is an assumption that an overload of dietary fatty acids leads to mitochondrial dysfunction in enterocytes and can increase intestinal permeability. To investigate this, the *in vitro* model of porcine enterocytes IPEC-J2 was incubated for 3 days with 250 µM lauric acid (C12:0), myristic acid (C14:0), palmitic acid (C16:0), or stearic acid (C18:0). Trans-epithelial electrical resistance, which indicates TJ integrity, showed that only C16:0 and C18:0 raised epithelial permeability with no impact on the expression of TJ protein genes. Bioenergetic analyses showed that C16:0 and C18:0 are scarcely β-oxidized. In contrast to C12:0 and C14:0, they caused a significant decoupling of OXPHOS and reduced ATP synthesis, which is associated with elevated generation of reactive oxygen species (ROS) in the mitochondria and mitochondrial splitting. Treatment with C12:0 and C14:0 resulted in significant lipid storage and increased mitochondrial network merging, but only slightly reduced ATP generation while leaving the epithelial barrier unchanged. In summary, these results suggest that the longer-chain fatty acids C16:0 and C18:0, in contrast to C12:0 and C14:0, enhanced intestinal permeability. Beyond that, only C16:0 and C18:0 caused significant energy depletion, particularly through elevated proton leakage, mitochondrial reshaping, and increased ROS in enterocytes.

The fourth original research article by Looi et al. deals with azithromycin (AZM), which is suspected of reducing the effects of human rhinovirus on the integrity and function of the barrier in healthy respiratory epithelium. Submerged and differentiated primary cultures of airway epithelial cells from healthy children were exposed to 10 µM AZM for 24 h followed by infection with human rhinovirus (HRV)-1b for 24 h. Subsequently, virus receptor expression, replication, progeny release, and inflammatory cytokines (IL-1β, IL−6, IL−8, and IP-10) were assessed in conjunction with barrier integrity. In uninfected cells treated with 10 µM AZM, no significant impact on the virus receptor and cytokine expression was noticed. Likewise, the expression of occludin and ZO-1 in non-infected cells remained unchanged. Claudin-1 gene expression was markedly decreased, while the protein expression was markedly elevated after 10 µM AZM. Even though the transepithelial electrical resistance was noticeably reduced, this change was not supported by a meaningful shift in epithelial permeability after exposure to 10 µM AZM. Following infection with HRV-1b, treatment with 10 µM AZM resulted in a significant decrease in cytotoxicity. The expression of viral receptors remained unchanged after pretreatment with AZM, but a significant decline in viral replication occurred. With the exception of IP-10, the expression of IL-1β, IL-6, and IL-8 was markedly decreased. Gene and protein expression of important epithelial junctions was considerably higher in treated, infected cells, along with the epithelial barrier performance. These findings demonstrate that AZM can prevent epithelial damage caused by HRV-1b. These results, which confirm the antiviral, anti-inflammatory, and barrier-protective effects *in vitro*, clearly point to multipurpose mechanisms of AZM for reducing viral infections along with their outcomes. Finally, these mechanisms are probably responsible for the clinical benefits of AZM seen in clinical trials for a range of chronic respiratory diseases.

The fifth original research article by DiGuilio et al. describes how Quercetin enhances and safeguards the barrier function of Calu-3 airway epithelia. Based on the evidence of respiratory barrier leakage in COVID-19 and the role of vitamin D in COVID-19 outcomes, especially in protecting the respiratory barrier, they explored whether the widespread dietary flavonoid quercetin might also be useful in maintaining respiratory barrier function. To tackle this question, they used the well-known Calu-3 airway epithelial cell culture model. Exposure of Calu-3 cell layers to quercetin raised the electrical resistance across the epithelium but at the same time decreased the transepithelial permeability of 14C-D-mannitol (Jm) and 14C-inulin. These actions of quercetin coincided with alterations in the composition of TJ protein and with partial suppression of cell replication that results in diminished linear junction density. These two effects can potentially help create a stronger barrier. The inhibitory effect of quercetin on DNA synthesis, which results in a reduction in cell replication in the Calu-3 cell layer, may also be a mechanism for enhancing barrier function, as it decreases the density of linear junctions. Quercetin proved similarly effective in mitigating the impairment of barrier function induced by the pro-inflammatory cytokine tumor-necrosis factor α (TNF-α), an effect that seemed to be partly due to the attenuation of the TNF-α-induced increase in Extracellular Signal-Regulated Kinases 1 und 2 (ERK1/2). In summary, quercetin enhanced the barrier function in Calu-3 and mitigated the TNF-α-triggered disruption of the barrier function, which was conveyed partly by alterations in the TJ complex.

All findings from the seven articles contribute to significantly increasing knowledge about the epithelial barrier, such as gastrointestinal and airway function. However, it still needs to be explored the different modes of impairments of the barrier, such as pore-route and leakage route and not simple the overall fluid flow through the barrier. Apart from genetic, epigenetic, molecular, and cell biological effects, the role of mechanobiology is still in its infancy and warrants future investigation.
